# Supplementary material for: Methodological Aspects of Green Extraction of Usnic Acid Using Natural Deep Eutectic Solvents
Source: Molecules. 2023 Jul 10;28(14):5321. doi: 10.3390/molecules28145321 (PMC10383951; doi:10.3390/molecules28145321)
Supplement: Supplementary file 1 [file molecules-28-05321-s001.zip › molecules-2462506-supplementary.pdf]

molecules

Article

Methodological Aspects of Green Extraction of Usnic Acid Using Natural Deep Eutectic Solvents

Magdalena Kulinowska [1], Sławomir Dresler [1,2], Agnieszka Skalska-Kamińska[1], Agnieszka Hanaka[2], and Maciej Strzemski[1,*]

1 Department of Analytical Chemistry, Medical University of Lublin, 20-093 Lublin, Poland
2 Department of Plant Physiology and Biophysics, Institute of Biological Sciences, Maria Curie-Skłodowska University, 20-033 Lublin, Poland
* Correspondence: maciej.strzemski@umlub.pl

Table S1. Usnic acid solubility in hydrophilic NADES at 20°C.

Natural Deep Eutectic Solvents	Molar ratio	Solubility (mg/100 ml)
**Proline-based**		
Proline : urea	1:1	13.30
Proline : levulinic acid	1:2	9.28
Proline : glycerol : saccharose	4:1:1	5.77
Proline : glycerol : saccharose	4:3:1	2.68
Proline : glycerol : saccharose	4:5:1	4.31
Proline : glycerol : saccharose	4:7:1	2.82
Proline : glycerol : saccharose	4:9:1	2.82
Proline : glycerol : saccharose	4:11:1	3.19
Proline : glycerol : saccharose	4:13:1	2.05
Proline : glycerol	1:1	4.59
Proline : glucose	1:1	2.90
Proline : tartaric acid : citric acid	1:1:1	1.85
Proline : fructose	1:1	1.31
Proline : citric acid	1:1	0.44
Proline : citric acid	1:2	0.34
**Urea-based**		
Urea : choline chloride	2:1	9.57
Urea : glycerol : citric acid	1:2:1	1.45
Urea : glycerol : citric acid	1:3:1	2.70
Urea : choline chloride : levulinic acid	1:1:1	0.74
**Betaine-based**		
Betaine : glycerol	1:2	5.01
Betaine : levulinic acid	1:2	3.74
Betaine : saccharose	4:1	1.22
Betaine : lactic acid : water	1:1.8:1	0.56
Betaine : glycerol : saccharose	4:5:1	0.31
**Choline chloride-based**		
Choline chloride : levulinic acid	1:2	3.55
Choline chloride : lactic acid : water	1:1.8:1	0.69
Choline chloride : lactic acid : water	1:2.7:1.5	1.92
Choline chloride : glycerol : saccharose	4:5:1	0.66
Choline chloride : citric acid : glycerol	1:1:1	0.60
Choline chloride : saccharose	4:1	0.29

Choline chloride : citric acid	1:1	0.08
Choline chloride : citric acid	2:1	0.03
Choline chloride : citric acid	3:1	0.02
Choline chloride : citric acid	1:2	0.16
Choline chloride : glycerol	1:2	0.14
Choline chloride : glycerol	1:1	0.13
Choline chloride : glucose	5:2	0.01
Choline chloride : glucose	2:1	0.01
Choline chloride : fructose	1:1	0.01
Choline chloride : fructose	5:2	0.02
**Glycerol-based**		
Glycerol : fructose	1:1	2.30
Glycerol : tartaric acid	1:1	2.03
Glycerol : citric acid	2:1	0.70
**Lactic acid-based**		
Lactic acid : water : glucose : fructose	0.9:0.5:1:1	1.83
Lactic acid : water : glucose	7.2:4:1	0.88
Lactic acid : water : glucose : citric acid	0.9:0.5:1:1	0.42
Lactic acid : water : β-alanine	0.9:0.5:1	0.33
**Citric acid-based**		
Citric acid : glucose	1:1	1.44
Citric acid : glucose	2:1	0.16
Citric acid : saccharose	1:1	0.65
Citric acid : β-alanine	1:1	0.35
